# S‐adenosylmethionine in combination with decitabine shows enhanced anti‐cancer effects in repressing breast cancer growth and metastasis

**DOI:** 10.1111/jcmm.15642

**Published:** 2020-07-28

**Authors:** Niaz Mahmood, Ani Arakelian, David Cheishvili, Moshe Szyf, Shafaat A. Rabbani

**Affiliations:** ^1^ Department of Medicine McGill University Health Centre Montréal QC Canada; ^2^ Department of Molecular Biology Ariel University Ariel Israel; ^3^ Gerald Bronfman Department of Oncology McGill University Montréal QC Canada; ^4^ Department of Pharmacology and Therapeutics McGill University Montréal QC Canada; ^5^ HKG Epitherapeutics Hong Kong China

**Keywords:** breast cancer, DNA methylation, epigenome, metastasis, S‐adenosylmethionine

## Abstract

Abnormal DNA methylation orchestrates many of the cancer‐related gene expression irregularities such as the inactivation of tumour suppressor genes through hypermethylation as well as activation of prometastatic genes through hypomethylation. The fact that DNA methylation abnormalities can be chemically reversed positions the DNA methylation machinery as an attractive target for anti‐cancer drug development. However, although in vitro studies suggested that targeting concordantly hypo‐ and hypermethylation is of benefit in suppressing both oncogenic and prometastatic functions of breast cancer cells, this has never been tested in a therapeutic setting in vivo. In this context, we investigated the combined therapeutic effects of an approved nutraceutical agent S‐adenosylmethionine (SAM) and FDA‐approved hypomethylating agent decitabine using the MDA‐MB‐231 xenograft model of breast cancer and found a pronounced reduction in mammary tumour volume and lung metastasis compared to the animals in the control and monotherapy treatment arms. Immunohistochemical assessment of the primary breast tumours showed a significantly reduced expression of proliferation (Ki‐67) and angiogenesis (CD31) markers following combination therapy as compared to the control group. Global transcriptome and methylome analyses have revealed that the combination therapy regulates genes from several key cancer‐related pathways that are abnormally expressed in breast tumours. To our knowledge, this is the first preclinical study demonstrating the anti‐cancer therapeutic potential of using a combination of methylating (SAM) and demethylating agent (decitabine) in vivo. Results from this study provide a molecularly founded rationale for clinically testing a combination of agents targeting the epigenome to reduce the morbidity and mortality from breast cancer.

## 1 INTRODUCTION

Abnormal DNA methylation is one of the earliest and most common hallmarks of cancer.^1^, ^2^ Since the addition or removal of the methyl group to the CpG islands is a dynamic and reversible process, it stands to reason that targeting the methylome may serve as an attractive anti‐cancer strategy.^3^, ^4^ Research over the past thirty years has led to the development of different types of DNA methylation inhibitors, and two drugs [5‐azacytidine (5AzaC, marketed as Vidaza) and 5‐aza‐2'‐deoxycytidine (5AzadC, marketed as decitabine/Dacogen)] targeting the DNA methyltransferase (DNMT) enzymes are already approved by the Food and Drug Administration (FDA) for the treatment of several haematological malignancies.^5^, ^6^, ^7^ Both 5AzaC and 5AzadC are cytosine analogues that can be incorporated into the DNA during replication where they function as suicide substrates for DNMT enzymes and trap them for subsequent proteasomal degradation, which ultimately leads to DNA demethylation at a genome‐wide scale.^8^, ^9^ At the molecular level, DNMT inhibitors (DNMTi) cause demethylation at the promoters of tumour suppressor genes that are otherwise methylated in cancer and thereby derepress their normal gene expression.^10^ More recently, it has been shown that DNMTi treatment also up‐regulates the expression of endogenous retroviral sequences (ERVs), which in turn activates viral defence response genes and thereby reduces the number of cancer‐initiating cells.^11^ These events reprogramme the cancer cells to behave similarly to the virus‐infected cells to cause the induction of an antitumour immune response against them, by a process known as ‘viral mimicry’.^10^

Even though DNMTis are approved for several haematological malignancies, they have only shown modest success in the case of solid tumours and generally induce toxic side effects like anaemia, bleeding and arthralgia.^12^ In addition, primary and secondary resistance to these drugs has been reported in clinical settings,^13^ which warrants the development of a different rational approach to target the methylome in solid cancers.

Since 5AzaC and 5AzadC induce demethylation across the genome, it stands to reason that the effect will not be limited to tumour suppressor genes and that they will also induce genes that promote cancer; notably genes involved in metastasis that are activated by loss of methylation. This might result in adverse effects and limit the utility of these agents. Indeed, it has been shown that DNMTi treatment also potentiates promotor demethylation‐mediated activation of several known prometastatic genes [urokinase plasminogen activator (*PLAU*), C‐X‐C motif chemokine receptor 4 (*CXCR4*), heparanase (*HPSE*)] in less aggressive MCF‐7 and ZR‐75‐1 breast cancer cells which facilitates their transformation to become more aggressive tumour cells.^14^ Interestingly, the treatment of cancer cells with universal methyl donor S‐adenosylmethionine (SAM) reverses these effects via hypermethylation of the promoters of the prometastatic genes.^15^ SAM is an approved nutraceutical agent used for osteoarthritis, fibromyalgia, cholestasis and depression,^16^, ^17^ and results from long‐term clinical trials showed no behavioural or biochemical adversities upon administration of the agent except for the individuals with bipolar disorder.^18^, ^19^ Our recent studies using xenograft models of breast cancer have demonstrated the anti‐cancer properties of SAM when administered via an oral route without causing any detrimental biochemical or behavioural adversities.^20^ Moreover, microarray‐based methylation studies on different types of cancer cells have revealed that SAM treatment caused hypermethylation‐mediated inactivation of prometastatic genes without repressing the expression of the known tumour suppressor genes.^21^, ^22^

Since DNA hypomethylation and hypermethylation are common characteristics of the cancer epigenome,^17^, ^23^ we have previously hypothesized and tested that combined administration of methylating and demethylating agents could block breast cancer growth and invasion in vitro.^24^ However, a critical question that remained unanswered was whether simultaneous targeting of DNA hypo‐ and hypermethylation using SAM and 5AzadC combination could show similar effects in vivo so that it could be further translated in clinical settings to breast cancer patients. Herein, we examined the anti‐cancer therapeutic potential of the approved demethylating agent 5AzadC in combination with global methyl‐group donor SAM in reducing tumour growth and metastasis using the MDA‐MB‐231 xenograft model of breast cancer.

## 2 MATERIALS AND METHODS

### 2.1 Cell culture and treatments

Human MDA‐MB‐231 (ATCC® HTB‐ 26™) and Hs578T *(*ATCC® HTB‐126*™*) triple‐negative breast cancer (TNBC) cell lines were maintained as described before.^20^ Authentication of both of these cell lines was done by short tandem repeat (STR) profiling using GenePrint® 10 System (Promega, Madison, WI, USA). DNA obtained from both cell lines showed a 100% match with the core alleles tested for authentication, which confirmed their identity. The mouse PyMT‐R221A breast cancer cell line was kindly provided by Dr Conor C. Lynch (H. Lee Moffitt Cancer Center and Research Institute, Tampa, FL, USA). These cells were initially extracted and cultured from the mammary tumours of transgenic MMTV‐PyMT mice in FVB background that resembles the luminal B subtype.^25^

The cells were treated with 200 μmol/L human‐grade SAM (a gift from Life Science Laboratories, Lakewood, NJ, USA), 1 μmol/L 5AzadC (Sigma‐Aldrich, St. Louis, MO, USA; Cat# A3656) or SAM + 5AzadC through direct administration of the agents into the culture medium every other day for six days as previously described.^24^

### 2.2 Cell proliferation and anchorage‐independent growth assay

The cells were seeded onto 6‐well cell culture grade plates (BD Falcon^TM^) and treated with SAM, 5AzadC, SAM + 5AzadC or vehicle (as control) every second day for six days. The coefficient of drug interaction (CDI) was measured to determine whether the interaction between the two drugs is synergistic, additive or antagonistic in different cell lines, as described before.^26^ On the day after each of the three treatments (on days 1, 3 and 5), the cells were trypsinized and counted using a Coulter counter (Model ZF; Coulter Electronics, Hertfordshire, UK). Following the usual treatment period, 5 × 10^3^ cells were used for anchorage‐independent growth assay as described before.^20^

### 2.3 Apoptosis assay

Following the usual treatment period stated above, both floating and adherent cells from vehicle‐treated control and different treatment groups were collected and subjected to Annexin V and propidium iodide staining using ‘Dead Cell Apoptosis Kit’ (Thermo Fisher, Eugene, OR, USA; Cat# V13242). As a positive control of apoptosis, the cells were treated with 20 μmol/L cisplatin (Abcam, Cambridge, UK; Cat# ab141398). Upon staining, the subsequent steps related to flow cytometry was performed as described before.^20^ The caspase‐3 enzyme activity was measured using the caspase‐3 assay kit (Abcam; Cat# ab39401) following the supplier provided protocol. The cell lysates from control and different treatment groups were incubated with DEVD‐*p*‐NA substrate containing buffer for 3 hours, and absorbance at 405 nm was measured by using a Tecan Infinite® 200 PRO microplate reader.

### 2.4 In vivo xenograft model of breast cancer

For in vivo experimental purpose, 6‐ to 8‐week‐old female immunocompromised NOD‐SCID mice were purchased from Charles River Laboratories (St‐Constant, QC, Canada) and housed at the Animal Resource Division (ARD) of the Research Institute of the McGill University Health Center (RI‐MUHC) at a 12‐hour light‐dark cycle in sterile cages with ad libitum access to food and water. After one week of acclimation in the RI‐MUHC ARD housing facility, animals were anaesthetized, and 5 × 10^5^ viable MDA‐MB‐231 cells mixed with 20% Matrigel (BD Biosciences) were inoculated into the fourth mammary fat pad of NOD‐SCID mice, as described by us previously.^27^ Three days after tumour cell inoculation, the animals were randomized and divided into four groups: vehicle [phosphate buffer saline (PBS)]‐treated controls, SAM (80 mg/kg/d) via oral gavage, 5AzadC (0.8 mg/kg/3 times per week for 3 weeks) by intraperitoneal (IP) injection and combination of SAM + 5AzadC. The doses used for the different agents were previously determined by us and others^20^, ^28^ and, therefore, the experimental protocol remained the same throughout the course of this study. Palpable tumours started to emerge from week 5 after inoculation, and the tumour volumes were measured at weekly intervals from week 6 until experimental endpoint on week 10 using the following formula: Volume = (length × Width^2^)/2. Relative tumour growth inhibition was measured using the following formula: 100*(1–*T_t_*/*T*_0_), where *T_t_* and *T_0_* stand to the mean tumour volumes for a treatment arm relative to the control arm.^29^

### 2.5 RNA extraction and qPCR

Total RNA was isolated using the AllPrep DNA/RNA Mini Kit (Qiagen, Hilden, Germany; Cat# 80 204) and converted to cDNA. Then, a quantitative PCR (qPCR) assay was performed using an ABI StepOnePlus™ (Applied Biosystems) machine following a previously described protocol.^21^ All primers used in this study are listed in Appendix S1: Table S1.

### 2.6 RNA‐Seq and analysis pipeline

Total RNA extracted from control, SAM‐, 5AzadC‐ and SAM + 5AzadC‐treated MDA‐MB‐231 cells was assessed by Agilent 2100 Bioanalyzer, and the samples that passed the quality control were used for transcriptome sequencing (n = 3/group). The supplier protocol for the NEBnext Ultra ii Stranded mRNA kit (New England Biolabs, Ipswich, MA, United States) was used for sample preparation, and an Illumina NextSeq 500 System was used for paired‐end sequencing. Once the sequencing was done, the alignment of the raw reads to the hg19 reference sequence (for *Homo sapiens*) was done using STAR aligners.^30^ Sequence assembly and differential gene expression analyses were done using the package Cufflinks.^31^ Differentially expressed genes from each treatment group relative to control MDA‐MB‐231 cells were chosen using a false discovery rate (FDR) adjusted *P*‐value of <0.2.

### 2.7 DNA isolation and MethylationEpic 850 K BeadChip microarray

DNA was extracted using the AllPrep DNA/RNA Mini Kit (Qiagen, Hilden, Germany; Cat# 80 204) following the standard protocol. Biological duplicates from each group were bisulphite‐converted, and epigenome‐wide methylation patterns were assessed using Infinium Human MethylationEpic 850K BeadChip microarray (Illumina) following the manufacturer's protocols. The Illumina intensity data (IDAT) files from the microarray experiment were normalized with BMIQ^32^ and processed using the ChAMP^33^ package as described by us before.^34^ The methylation levels were obtained as *β* values that ranged from zero to one (‘0’ = fully unmethylated probe and ‘1’ = fully methylated probe). Probes with single nucleotide polymorphism (SNP) were removed from the downstream analysis. For differential methylation analysis in each treatment group relative to controls, Bioconductor package Limma^35^ was used where a methylation difference (delta *β* value) >0.05^36^ and *P* ˂ .05 was considered statistically significant as previously described.^37^

### 2.8 Western blot

Cell lysates were prepared using radioimmunoprecipitation assay (RIPA) buffer containing a mixture of protease and phosphatase inhibitors, and Western blot was done as described by us previously.^38^

### 2.9 Immunohistochemistry

The immunohistochemical assessment was performed on formalin‐fixed tumour tissues by double staining each sample slide using antibodies against Ki‐67 (Cat# M7240, Dako, Glostrup, Denmark) and CD31 (Cat# 760‐4378, Roche, Basel, Switzerland) markers. The staining was done at the RI‐MUHC Histopathology platform using a standardized protocol. Then, photomicrographs of five randomly selected fields from each sample slides were taken. The Ki‐67‐positive cells were counted based on their distinct nuclear staining. The area of CD31 staining was quantified using ImageJ (Fiji plugin) (National Institutes of Health, Bethesda, MD, USA).

### 2.10 Statistical analysis

The results are expressed as the mean ± standard error of the mean (SEM). Statistical analyses were carried out by Student's *t* test, ANOVA depending on the type of experimental data. *P* ≤ .05 was considered as significant.

## 3 RESULTS

### 3.1 Effect of SAM + 5AzadC combination on TNBC cell lines in vitro

**FIGURE 1:**
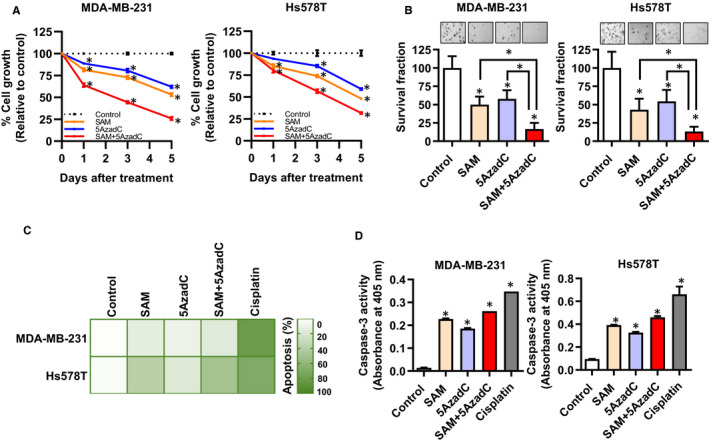
Effect of SAM, 5AzadC and their combination on cell proliferation, colony formation and apoptosis in vitro. (A) MDA‐MB‐231 and Hs578T were treated with vehicle only (as control), SAM (200.0 µmol/L), 5AzadC (1.0 µmol/L) and SAM + 5AzadC every second day for a period of six days, and the percentage change in cell proliferation relative to the control group at different time‐points is shown as line graphs. (B) The total number of colonies in each treatment group was directly counted under a light microscope and plotted as bar graphs. (C) Heatmap showing the average levels of apoptosis in control and different treatment groups. As a positive control for apoptosis, both cell lines were treated with 20 µmol/L cisplatin. (D) Caspase‐3 enzyme activity was measured from the cell lysates of control and different treatment groups. Results are represented as the mean ± SEM. Statistical analysis was done using ANOVA followed by post hoc Tukey's test, and significant differences are shown by asterisks (*P < 0.05)

To examine the in vitro effect of combining SAM and 5azadC on the growth properties of cells, we first used two highly metastatic TNBC cell lines: MDA‐MB‐231 and Hs578T. Both SAM and 5AzadC were previously shown to reduce tumour cell proliferation.^21^, ^22^, ^39^ As expected, either 200 µmol/L SAM or 1 μmol/L 5AzadC caused a significant reduction in cell growth compared to vehicle‐treated controls (Figure 1A). However, combination therapy caused a more substantial reduction in growth than either of the monotherapies. A coefficient of drug interaction (CDI) test using cell proliferation data showed a moderately synergistic effect of the combination treatment in MDA‐MB‐231 (CDI = 0.78) cells and an additive effect in Hs578T cells (CDI = 1.1) in vitro.

Next, we evaluated whether the combination of SAM and 5AzadC could suppress the anchorage‐independent growth, a cellular measure of malignant transformation. A significant decrease in the ability of the MDA‐MB‐231 and Hs578T cells to form colonies was observed upon single‐agent treatment with either SAM or 5AzadC (Figure 1B). The suppression was significantly more pronounced when the cells were treated with the combination of SAM and 5AzadC in both cell lines (Figure 1B).

Next, we wanted to evaluate whether the combination treatment shows a similar anti‐cancer effect in breast cancer cells belonging to a different subtype and species. For that, we used the PyMT‐R221A murine luminal B breast cancer cell line. Our data showed a moderately synergistic effect of the combination (CDI = 0.75) in decreasing PyMT‐R221A cell proliferation (Appendix S1: Figure S1A). A significant reduction in the ability of the PyMT‐R221A cells to form colonies was also observed (Appendix S1: Figure S1B). Taken together, these observations suggest that the combination treatment inhibits the growth of a broad spectrum of breast cancer cells representing different subtypes and species.

We then examined the effect of the combination treatment on apoptotic cell death using a flow cytometry‐based annexin V/PI assay. While all three treatment groups induced apoptosis, the SAM + 5AzadC‐treated cells showed the highest percentage of apoptotic cell deaths (Figure 1C). We used the DNA‐damaging agent cisplatin as a positive control for the induction of apoptosis. To further confirm these results, we measured the enzymatic activity of caspase‐3, which functions as an executioner caspase to induce apoptosis.^40^ A significant increase in caspase‐3 activation was observed in the treated groups compared to the control MDA‐MB‐231 and Hs578T cells (Figure 1D). During these studies, we did not observe any noticeable change in the morphology of the cells.

### 3.2 Effect of SAM + 5AzadC combination on MDA‐MB‐231 xenograft model of breast cancer

**FIGURE 2:**
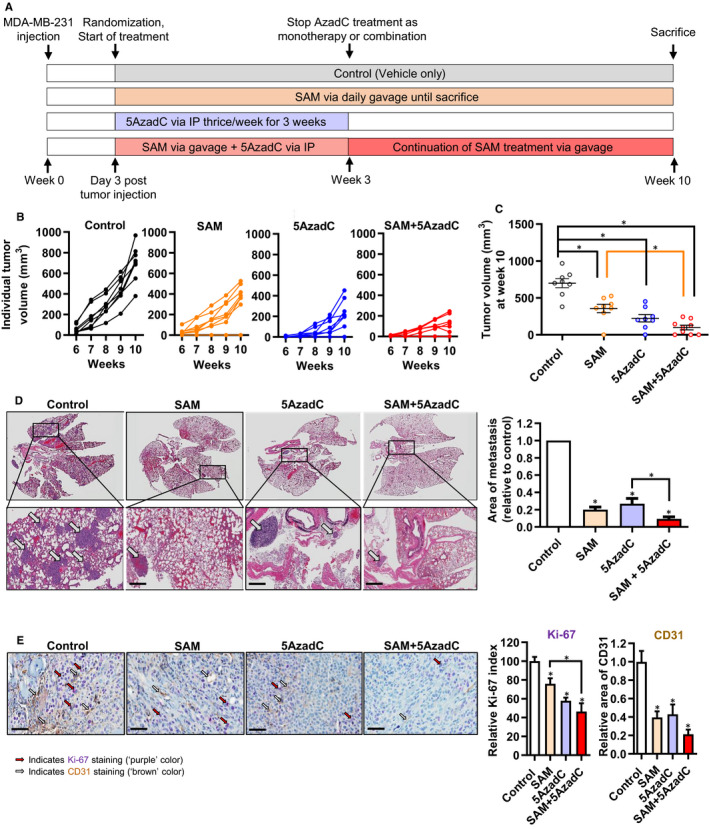
Effect of SAM, 5AzadC and their combination on MDA‐MB‐231 xenograft tumour growth and lung metastasis. (A) Schematic of the treatment protocol. (B) The tumour growth kinetics of individual animals from each group were plotted as line graphs. (C) The average tumour volume in each group of animals at experimental endpoint (10 wk after the initial injection of tumour cells). Results are represented as the mean ± SEM of at least eight animals in each group (*P < 0.05). (D) Representative photomicrographs of whole lung sections from each group (top panel). The bottom panel shows a higher magnification image from a region of micrometastases [scale bar size = 500 μm]. The average areas of metastases were plotted as a bar graph (n = 3 animals/group). (E) Formalin‐fixed primary breast tumours were double immunostained with Ki‐67 and CD31 antibodies (n = 3 animals/group). The photomicrograph of five randomly selected fields from each sample was analysed and represented as bar graphs. Results are shown as the mean ± SEM. Statistical analysis was done using ANOVA followed by post hoc Tukey's test, and significant differences are shown by asterisks (*P < 0.05)

We then tested the anti‐cancer therapeutic effect of SAM combined with 5AzadC in vivo using a human MDA‐MB‐231 xenograft model of TNBC, where 5 × 10^5^ tumour cells were orthotopically implanted into the fourth inguinal mammary fat pads of 6‐ to 8‐week‐old female NOD‐SCID mice. Three days post‐injection of the tumour cells, the animals were randomized into four groups: PBS vehicle‐treated controls, 80 mg/kg/d of SAM via oral gavage, 0.8 mg/kg of 5AzadC by IP injection and combination [SAM (80 mg/kg/d)+5AzadC (0.8 mg/kg)], and treatment was carried out using the strategy depicted in Figure 2A. The 5AzadC treatment, for the monotherapy and combination groups, was carried out for three weeks to avoid potential adverse effects.^12^ In contrast, all animals treated with SAM in the monotherapy and the combination setting received SAM daily via oral gavage from the start of treatment until the experimental end‐point was reached. Our results show that all animals in the control group developed tumours that continued to grow until the experimental end‐point at week 10 after tumour cell injection (Figure 2B; Appendix S1: Figure S2). On the other hand, 87.5% of the animals in either SAM or 5AzadC monotherapy treatment groups developed a tumour at the experimental end‐point, while only 66.67% of the animals in the SAM + 5AzadC combination treatment group developed a tumour (Appendix S1: Figure S1). Moreover, in comparison with the control animals, significant reductions in tumour volumes were observed in the treatment groups at experimental endpoint on week 10 (Figure 2B‐C). To determine whether the anti‐cancer therapeutic effects in the combination treatment are either additive, synergistic or antagonistic, we measured CDI and found that the combination treatment shows a moderately synergistic effect (CDI = 0.86) in reducing the primary mammary tumour volumes in this model. We also measured tumour growth inhibition at experimental endpoint in each group relative to control animals and found 49.13% and 67.95% reduction in average tumour volume in SAM‐ and 5AzadC‐treated animals, respectively. However, the reduction in tumour volume in the combination treatment group was 85.96% relative to the controls suggesting an enhanced anti‐cancer activity of the combination treatment in reducing mammary tumour growth in vivo.

Since MDA‐MB‐231 cells orthotopically implanted into the mouse mammary fat pad metastasize to different target organs,^20^ we evaluated the effect of the different treatment regimens on lung metastasis using H&E staining of formalin‐fixed lung tissue sections from the different treatment groups. Compared to the vehicle‐treated control animals, a significant reduction in lung metastasis was observed in both SAM and 5AzadC monotherapy‐treated animals, which was further reduced in the SAM + 5AzadC‐treated animals (Figure 2D), demonstrating a higher anti‐cancer therapeutic effect of the combination treatment.

The aggressiveness of breast cancer correlates with proliferative capabilities as well as the vascularization of the tumour cells, which prompted us to determine the expression of Ki‐67 (proliferation marker) and CD31 (angiogenesis marker) in formalin‐fixed tumour tissues from control and the treatment groups using a double immunostaining strategy. Our data showed that animals from all three treatment groups had a significant reduction in the expression of proliferation and angiogenesis markers, an effect that was more pronounced in the SAM + 5AzadC combination‐treated group (Figure 2E). Taken together, these results complement the phenotypic effect seen by the reduced tumour volume and metastasis in response to the combination treatment.

Next, we checked whether the SAM + 5AzadC combination treatment elicits any toxicities in the animals by measuring different biochemical parameters related to liver and kidney function as well as major electrolytes. Our data demonstrated that there were no statistically significant differences in any of the blood parameters tested between control and the treatment arms (Appendix S1: Table S2), suggesting that the treatments are not overtly toxic.

### 3.3 Effect of SAM + 5AzadC combination on the MDA‐MB‐231 transcriptome

**FIGURE 3:**
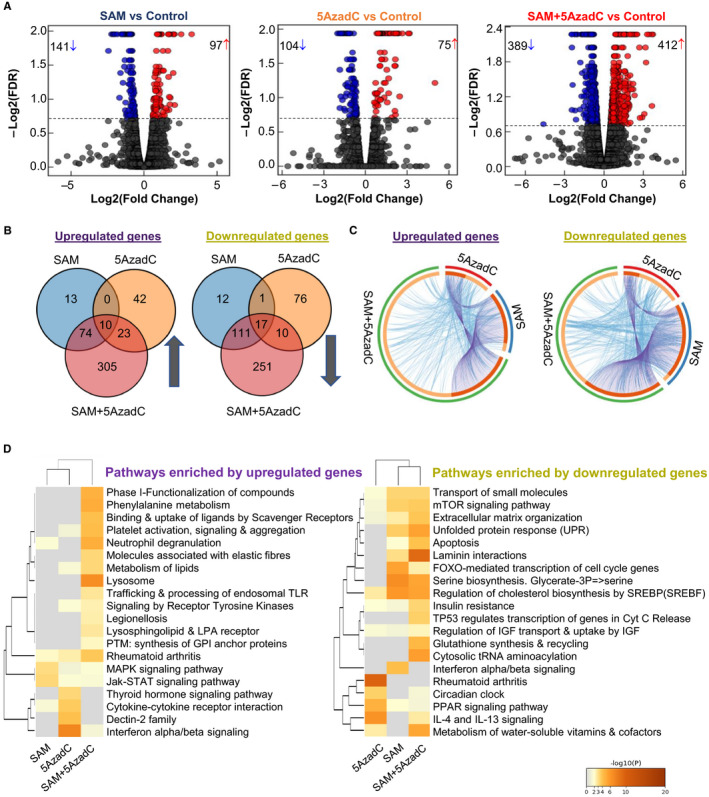
Transcriptome analyses of MDA‐MB‐231 cells. (A) The volcano plot of the DEGs obtained from global transcriptome analyses in each treatment group relative to vehicle‐treated control MDA‐MB‐231 cells is shown (n = 3/group). (B) Venn diagrams representing the overlap of DEGs among different treatment groups. The up‐regulated and down‐regulated genes were analysed and represented separately. (C) The circos plot depicting the functional overlap between the up‐ and down‐regulated DEGs in each treatment group. On the arc, there is a spot for each of the genes showing significant down‐ or up‐regulation (for all three treatment groups). The darker orange indicates the genes common in multiple groups, while the lighter orange indicates the unique genes for a particular treatment. The purple (criss‐crossed) lines represent the genes that are common in different groups, while the blue lines are given for the genes with similar functions. (D) Comparative heatmap of the pathways enriched by the up‐ and down‐regulated genes in different treatment groups, as determined by Metascape^42^

To evaluate gene expression changes mediated by different treatments, we next performed a transcriptome analysis of the control and treated MDA‐MB‐231 cells by RNA‐sequencing (n = 3/group). Our data revealed that, in comparison with the vehicle‐treated control MDA‐MB‐231 cells, single‐agent treatment with SAM and 5AzadC caused significant gene expression changes of 238 (141 down‐regulated, 97 up‐regulated) and 179 (104 down‐regulated, 75 up‐regulated) genes, respectively (Figure 3A, Appendix S2). Interestingly, these effects were more pronounced in the SAM + 5AzadC combination‐treated cells, where 801 (389 down‐regulated, 412 up‐regulated) genes were differentially expressed relative to the control (Figure 3A, Appendix S2). We then used Venn diagrams and circus plots to depict the numeric and functional common and exclusive transcriptomic footprints in the different treatment groups. Our analyses indicated that the combination therapy significantly changes the expression profiles of 556 genes (305 up‐regulated and 251 down‐regulated) that are not significantly affected by either of the monotherapy treatments using our study cut‐offs (Figure 3B, C).

We then performed comparative pathway enrichment analyses between differentially expressed genes (DEGs) in SAM‐, 5AzadC‐ and SAM + 5AzadC‐treated cells using the well‐annotated Reactome and Kyoto Encyclopedia of Genes and Genomes (KEGG) databases from Metascape^41^ (Figure 3D). We found that the genes down‐regulated by the combination treatment are enriched in ‘Laminin interactions’, ‘Extracellular matrix organization’ pathways that are involved in migration, invasion, and metastatic spread, while the genes up‐regulated upon the combination treatment are enriched in crucial cancer‐related pathways like ‘Interferon alpha‐beta signalling’, ‘Jak‐STAT signalling pathway’ and others as shown by the heatmap in Figure 3D.

### 3.4 Validation of differentially expressed cancer‐related genes affected by the combination treatment in MDA‐MB‐231

**FIGURE 4:**
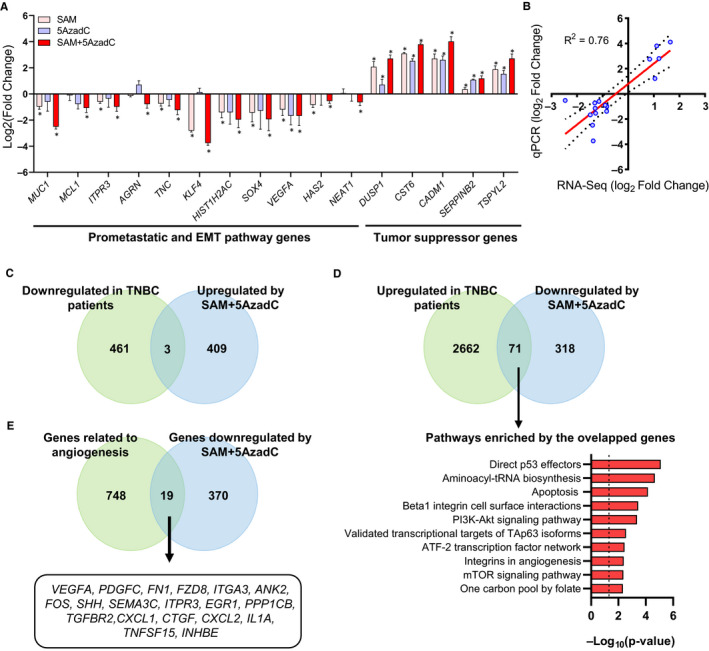
Validation of selected genes that showed significant differential expression following SAM + 5AzadC combination treatment. (A) qPCR validation of selected cancer‐related genes obtained from RNA‐sequencing. Results are represented as the mean ± SEM. (*P < 0.05; n = 3/group). (B) The correlation coefficient between RNA‐Seq (in x‐axis) and qPCR (in y‐axis) data [presented as Log_2_(Fold Change)] was performed using a Pearson test was found to be 0.76 with a *P* < 0.001. (C‐D) Comparison of the significantly up‐ or down‐regulated genes upon SAM + 5AzadC treatment with differentially expressed genes obtained from the transcriptome analyses of TNBC patients showed an overlap of 3 and 71 genes, respectively. A pathway analysis [bottom panel of (D)] of 71 overlapped genes that were up‐regulated in TNBC but down‐regulated in the combination showed involvement of molecular signatures related to cancer growth, metastasis and apoptosis. (E) A comparison of the significantly down‐regulated genes upon SAM + 5AzadC treatment with the genes involved in angiogenesis (from the Metascape database) showed a significant overlap of 19 genes

We next validated the differential expression of several prometastatic and tumour suppressor genes that are involved in various cancer‐related signalling pathways in the treatment groups by quantitative polymerase chain reaction (qPCR) analysis (Figure 4A). There was a significant correlation between fold change in expression determined by RNA‐Seq and by qPCR between combination treatment and control (Figure 4B).

Recent evidence suggests that suppression of MUC1, in turn, down‐regulates the anti‐apoptotic MCL1 protein in breast cancer cells.^42^ Interestingly, *MCL1* gene expression is reduced upon SAM + 5AzadC, as shown by RNA‐Seq (Appendix S2) and qPCR (Figure 4A). We then measured the levels of the anti‐apoptotic proteins MCl‐1 and BCl‐2 and found that they were reduced in response to the combination treatment (Appendix S1: Figure S3A). Previous studies have demonstrated that increased MUC1 expression stabilized beta‐catenin from degradation by glycogen synthase kinase 3 beta (GSK3B).^43^ Therefore, we measured the expression of β‐catenin, which is a component of the pro‐proliferative Wnt/β‐catenin signalling pathway and found it to be down‐regulated upon SAM + 5AzadC treatment (Appendix S1: Figure S3A). These results indicate that the combination treatment affects multiple components of pro‐proliferative and anti‐apoptotic pathways to elicit an anti‐cancer response (Appendix S1: Figure S3B).

We next used publicly available cancer transcriptome data set (as described by Solzak et al^44^) to determine whether the genes targeted by the combination treatments (DEGs) are known to be differentially expressed in triple‐negative breast cancer patients and therefore potentially important for the cancer state. Although the overlap between the genes which were up‐regulated by SAM + 5AzadC but down‐regulated in patients was not significant as determined by a hypergeometric test (Figure 4C), there was a significant overlap of 71 genes which were down‐regulated by SAM + 5AzadC treatment with the set of genes which were up‐regulated in breast cancer patients (hypergeometric test, *P* < 0.05) (Figure 4D). These data point to a potential benefit of SAM + 5AzadC treatment for highly aggressive TNBC patients. Pathway enrichment analysis of these genes revealed that they are involved in several cancer‐related signalling pathways like the p53 downstream pathway, apoptosis, Beta1 integrin cell surface interactions and PI3K signalling pathways (Figure 4D), suggesting the clinical relevance of the genes differentially regulated by SAM + 5AzadC combination.

We then assessed whether the phenotypic changes related to metastasis and angiogenesis seen in vivo could be linked to the gene expression changes induced by the SAM + 5AzadC combination treatment in vitro. We first overlapped the genes down‐regulated by the combination treatment with the complete repertoire of metastatic genes obtained from the human cancer metastasis database^45^ and found a significant overlap of 66 genes (hypergeometric test, *P* < 0.05) (Appendix S1: Figure S4). We then compared the genes down‐regulated by the SAM + 5AzadC combination with the list of genes involved in angiogenesis and found a statistically significant overlap of 19 genes (hypergeometric test, *P* < 0.05) (Figure 4E). Some of the crucial genes in this overlap include *VEGFA, PDGFC and FN1* that were known to be involved in angiogenesis in different types of cancers. Taken together, these results suggest that the transcriptome‐wide gene expression changes show congruence with the phenotypic changes mediated by the SAM + 5AzadC combination.

### 3.5 Effect of the SAM + 5AzadC combination on the upstream regulators of gene expression

**FIGURE 5:**
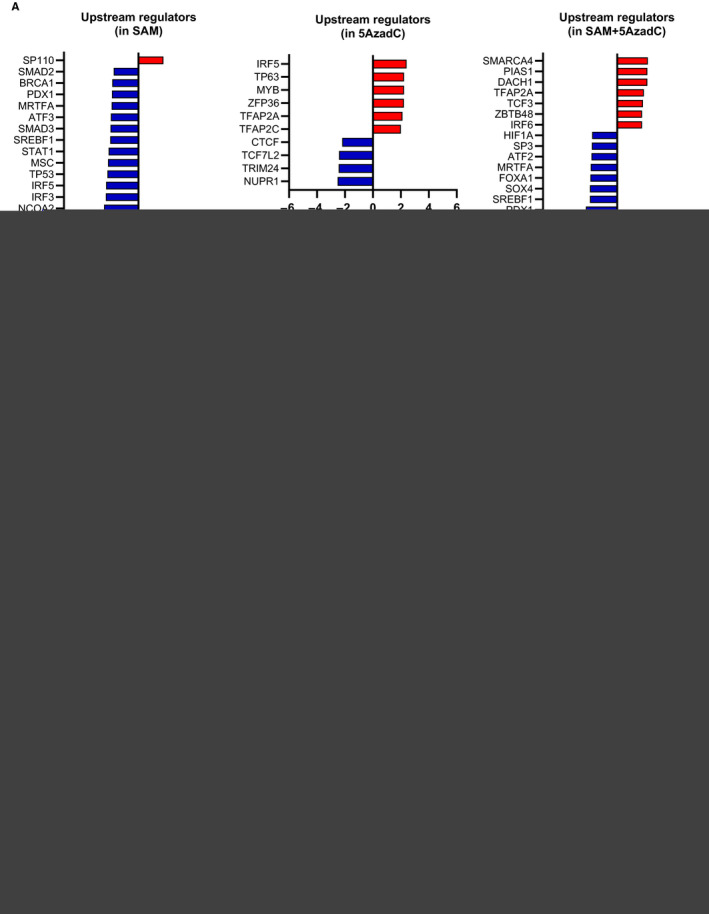
Upstream transcription regulator analyses. (A) IPA tool predicted list of significantly activated and inhibited upstream transcription regulators in each treatment group is shown as bar graphs. A z‐score greater than 2.0 defines significant activation of the node, whereas a z‐score less than 2.0 defines inhibition. (B) HIF1A and SOX4 are among the several transcription regulators predicted to be inhibited by SAM + 5AzadC treatment. Target molecules of HIF1A and SOX4 from the list of DEGs upon SAM + 5AzadC treatment are shown. (C) Mechanistic pathway analyses of HIF1A and SOX4, according to the IPA knowledge base, show the network of molecular targets that are possibly affected by the combination treatment

To identify the potential upstream regulators that mediate the gene expression changes seen in RNA‐Seq, an upstream regulator analysis (URA) was performed using ingenuity pathway analysis (IPA) tool.^46^ This analysis can decipher the potential transcription regulators, growth factors and any gene or chemical that has been shown to affect gene expression by experimental evidence. We mainly focused on the ‘transcription regulators’ that directly regulate gene expression. Our results show that a total of 16 (1 up, 15 down), 10 (6 up, 4 down) and 18 (7 up, 11 down) transcription regulators are predicted to be significantly affecting the DEGs enriched in SAM, 5AzadC and SAM + 5AzadC combination‐treated groups, respectively (Figure 5A). The upstream transcription regulators that were activated in SAM + 5AzadC were TFAP2A, PIAS1, ZBTB48, TCF3, DACH1, SMARCA4 and IRF6 which affect a diverse array of target genes that are graphically depicted in Appendix S1: Figure S5. For example, PIAS1 activation might repress *MCL‐1* expression, whereas TFAP2A activation might down‐regulate *VEGFA*, *KLF4* and several other genes, as seen in the RNA‐Seq data. When we investigated the upstream transcription regulators that were inhibited upon SAM + 5AzadC treatment, we found a significant change in some of the well‐known cancer‐related transcription factors like HIF1A and SOX4 (Figure 5A), whose downstream target genes are shown in Figure 5B. The genes targeted by the other upstream regulators inhibited by SAM + 5AzadC treatment are presented in Appendix S1: Figure S6. We next focused on HIF1A and SOX4 mediated effects through the construction of mechanistic networks using the existing knowledge found in the IPA tool (Figure 5C). The mechanistic network analyses suggest that the inhibition of SOX4 possibly mediates HIF1A down‐regulation which, in turn, affects various downstream oncogenic factors, as shown in Figure 5C. These observations indicate that the SAM + 5AzadC treatment alters the expression of crucial transcription factors that mediate the downstream changes in expression of a vast array of genes in the MDA‐MB‐231 transcriptome.

### 3.6 Effect of SAM + 5AzadC combination on the MDA‐MB‐231 methylome

**FIGURE 6:**
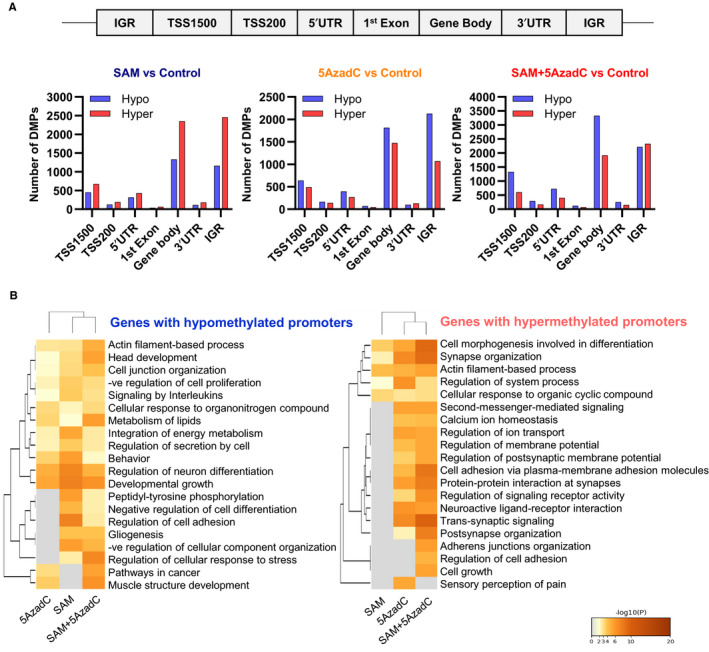
Methylome analyses of MDA‐MB‐231 cells. (A) Genome‐wide distribution of the differentially methylated CGs from each treatment (n = 2/group). Here, UTR: untranslated region; IGR: intergenic region; and TSS: transcription start site. (B) Pathway enrichment analyses of the genes associated with differentially methylated probes near the promoter regions following treatments with SAM, 5AzadC and SAM + 5AzadC

Since both SAM and 5AzadC modulate DNA methylation, we next used a genome‐wide approach to delineate the changes in MDA‐MB‐231 methylome in response to treatment with either single‐agent therapies or the combination using Illumina MethylationEPIC arrays that cover more than 850k probes. In this study, we defined probes with >5% change in methylation in both directions in treatment compared to control groups as either hypermethylated or hypomethylated, respectively. As expected, we found that SAM monotherapy caused more hypermethylation, while 5AzadC monotherapy caused more hypomethylation of CpG sites (Figure 6A). The combination treatment caused broader changes in the DNA methylation landscape than the monotherapy treatments by either SAM or 5AzadC; DNA methylation changes happened in both directions at different locations in the genome (Figure 6A). The combination treatment caused more hypomethylation near the promoter regions (TSS1500, TSS200 and 5′UTR as defined in Ref^47^) and slightly more hypermethylation in the IGRs.

Differential methylation at the promoter region of genes is believed to mediate tumorigenesis either via the down‐regulation of tumour suppressor genes or up‐regulation of oncogenes. We then focused on the CpGs that are differentially regulated by the SAM + 5AzadC combination treatment at or near promoter regions. Using a gene set enrichment analysis, we found that combination treatment causes significant methylation changes of genes involved in several crucial cancer‐related pathways (Figure 6B). For example, we found that the genes whose promoters become hypomethylated upon combination treatment are enriched in pathways that are involved in ‘negative regulation of cell proliferation’, while the genes whose promoters are hypermethylated are engaged in ‘cell proliferation’ (Figure 6B). Taken together, these observations further validate that epigenetic therapies with SAM and 5AzadC alter DNA methylation of critical cancer‐related pathways. It should also be noted that the methylation changes have context‐dependent roles in regulating gene expression, and not all the methylation changes will result in altered gene expression.

### 3.7 Integrated analyses of methylation and gene expression in the combination therapy group

**FIGURE 7:**
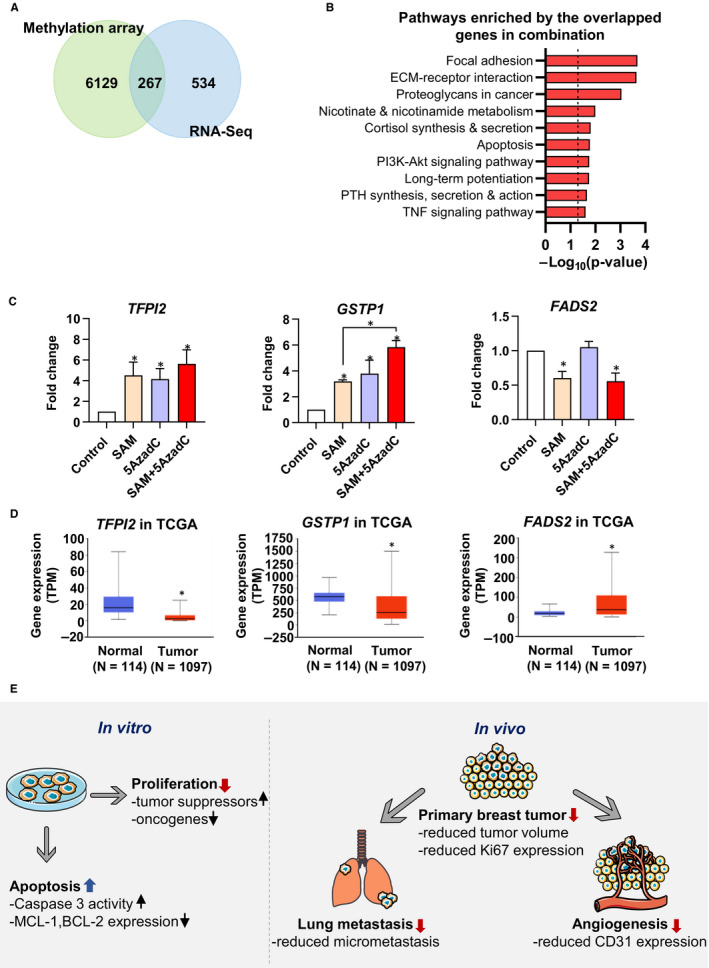
Integrative analyses of MD‐MB‐231 methylome and transcriptome. (A) Venn diagram depicting the overlap of genes that showed hypomethylation‐mediated inactivation and hypermethylation‐mediated activation upon the combination therapy treatment in genome‐wide methylation and RNA‐Seq data sets. (B) Pathways enriched by the overlapping genes from the methylation array and RNA‐Seq. (C) qPCR validation of several overlapped genes (*TFPI2*, *GSTP1* and *FADS2*) in response to the indicated treatments. Results are shown as the mean ± SEM. (*P < 0.05; n = 3/group). (D) The expression of *TFPI2* and *GSTP1* genes is down‐regulated, while *FADS2* expression is up‐regulated in the TCGA transcriptome data sets of breast cancer patients. (E) Schematic summarization of the SAM + 5AzadC mediated anti‐cancer effects, according to the results of this study

To gain further molecular mechanistic insights to the set of genes regulated by the combination therapy, we determined whether the changes seen in DNA methylation were associated with changes in gene expression as determined by RNA‐Seq. The integrated analyses of transcriptome and methylome showed that differential expression of 267 genes was associated with differential DNA methylation (Figure 7A). Further analysis revealed that these genes are enriched in cancer‐related signalling pathways like focal adhesion, ECM‐receptor interaction, apoptosis, PI3K‐AKT signalling and others as listed in Figure 7B. In addition, we found that 60 out of these 267 genes showed a significant overlap with the list of genes obtained from the human cancer metastasis database (hypergeometric test, *P* < 0.05) (Appendix S1: Figure S7).

Next, from the list of 267 overlapping genes between expression and methylation, we focused on genes whose promoter was hypermethylated with down‐regulated gene expression and genes whose promoters were hypomethylated with up‐regulated gene expression. Through integrated analyses of gene expression and promoter methylation, we identified 45 genes that showed hypomethylation‐mediated up‐regulation and 15 genes that showed hypermethylation‐mediated down‐regulation upon SAM + 5AzadC combination treatment (Appendix S1: Table S3). We then validated the expression of the several genes from the list Appendix S1: Table S3 that showed hypomethylation‐mediated activation (*TFPI2* and *GSTP1*) and hypermethylation‐mediated inactivation (*FADS2*) upon combination treatment by qPCR (Figure 7C). Further analysis of the Cancer Genome Atlas (TCGA) database suggested that these genes show aberrant expression patterns in breast cancer patients in the opposite direction (Figure 7D).

Taken together, through a series of in vitro and in vivo studies, we demonstrated a higher anti‐cancer effect of the SAM + 5AzadC combination in comparison with monotherapies in well‐established models of breast cancer and elucidated its potential molecular basis (Figure 7E).

## 4 DISCUSSION

Epigenetic reprogramming in cancer involves a combination of demethylation‐mediated activation of tumour‐promoting and prometastatic gene networks and hypermethylation‐mediated silencing of tumour suppressor gene networks. Currently, Vidaza and decitabine are the only approved DNA methylation inhibitors used clinically to treat cancer patients. However, a long line of evidence has established in cell culture studies that loss of methylation can lead to the induction of genes that promote metastasis, the most morbid facet of cancer. Several studies showed that demethylating agents can enhance the invasiveness of breast cancer cells in vitro^14^, ^39^ and that the methyl donor SAM could inhibit invasiveness and bone metastasis in vivo.^15^, ^21^ One possible way to attenuate the adverse effects of DNA demethylation agents is to enhance methylation of tumour and metastasis promoting genes using the ubiquitous methyl donor SAM. This suggestion is presumably counterintuitive however, since the addition of a methylation promoting agent such as SAM might cancel the activation effect of 5AzadC on tumour suppressor genes and thus both agents will nullify each other and eliminate the therapeutic effect. However, if SAM targets different gene pathways than 5AzadC, the combination could be synergistic. A rational approach to cancer therapy should involve a combinatorial approach targeting different nodal pathways of growth and metastasis concurrently. A previous in vitro study confirmed that a combination of SAM and 5AzadC would be efficacious and, more importantly, inhibit metastasis which is stimulated by 5AzadC.^24^ Could this be translated into clinical practice? A first step should be demonstrating that a combination of SAM and 5AzadC will have more efficacious anti‐cancer activity than monotherapies in vivo and second that this combination inhibits cancer metastasis in vivo.

In this study, we compared SAM + 5AzadC combination therapy with monotherapies with either compound using a well‐established in vivo model of breast cancer. Even though the combination treatment shows anti‐cancer effects on breast cancer cells from different subtypes, we focused on TNBC due to the high rate of mortality in patients with this breast cancer subtype and a paucity of effective therapeutic strategies. Our data showed that the combination of SAM + 5AzadC had a moderately synergistic anti‐cancer effect on reducing primary tumour volumes of MDA‐MB‐231 xenografts without causing additional toxicity as measured by standard biochemical tests. Moreover, the metastatic spread of primary tumour cells from the breast to the lung tissue was robustly inhibited by combination therapy as compared to monotherapy with 5AzadC. These data support the conclusion that a combination of 5AzadC and SAM might be of utility in treating breast cancer and potentially other cancers and warrant further clinical testing. Our results show that SAM does not nullify the effects of 5AzadC, but it rather enhances the antitumour effect.

To examine the molecular mechanism underlying enhanced anti‐cancer potential of the combination therapy, and to test whether combination therapy interferes with the effect of DNA demethylation on the induction of tumour suppressor genes, we performed genome‐wide methylome and transcriptome analyses following treatment with SAM, 5AzadC and their combination. Results from these studies demonstrate that the two agents (SAM and 5AzadC) target a diverse array of genes acting in different functional pathways involved in cancer development and progression, explaining why SAM does not nullify the effects of 5AzadC. The combination therapy did not block 5AzadC activation of tumour suppressor genes and did not result in the silencing of other tumour suppressor genes. On the contrary, the combination treatment up‐regulated expression of several known tumour suppressor genes like *CST6*, *TFPI2*, *GSTP1* and several others.

The combination treatment showed enhanced anti‐cancer effects in reducing the expression of genes related to metastasis compared to the monotherapy treatment. For example, the expression of *Sox4*, a master regulator of EMT,^48^ was significantly reduced in the combination group suggesting the possible modulation of the treatment through the pathway. We also found that the expression of the *MUC1* gene, which is overexpressed in breast cancer patients (Appendix S1: Figure S8), and encodes the widely used CA 15‐3 (Cancer antigen 15‐3) serum biomarker for breast cancer, was significantly reduced upon SAM + 5AzadC combination treatment. MUC1 promotes cancer cell invasion and epithelial‐to‐mesenchymal transition (EMT) through its interaction with beta‐catenin,^49^ both of which are reduced upon treatment with SAM + 5AzadC (Figure 4A; Appendix S1: Figure S2). The expression of *KLF4*, required for breast cancer stem cell (CSC) maintenance,^50^ was also significantly repressed upon combination treatment (Figure 4A). This prompted us to check whether any other critical modulators of the pathways related to CSC were altered by the combination treatment and found that the expression of *SHH*, which is up‐regulated in human breast tumours (Appendix S1: Figure S9A), is down‐regulated by the combination treatment (Appendix S1: Figure S9B).

Only 27 DEGs (10 up‐regulated and 17 down‐regulated) were found to be commonly targeted by SAM‐, 5AzadC‐ and SAM + 5AzadC‐treated breast cancer cells in RNA‐Seq. The combination‐treated cells shared more DEGs with SAM monotherapy (185 common genes)‐treated cells than 5AzadC monotherapy (33 common genes)‐treated cells. Importantly, the combination therapy targeted 556 genes that were not targeted by either agent on its own, and these genes involved in pathways related to cancer growth and metastasis (Appendix S1: Figure S10). Thus, the molecular footprint of the combination therapy is not just an additive combination of either monotherapy; it affects hundreds of genes that would not be affected by either monotherapy suggesting a synergism between these two epigenetic modulators. These data provide a molecular rationale for combining these agents in the clinical setting.

Most phase I clinical trials using DNMTis for the treatment of solid tumours have not been successful,^51^ possibly due to the relatively short half‐life of the agent as well as susceptibility to deamination and subsequent inactivation.^52^, ^53^ Hence, combining 5AzadC with other anti‐cancer agents was proposed in the case of solid cancers.^52^ Our data provide a different mechanism to counteract the adverse effects of 5AzadC monotherapy by using a unique molecularly and preclinically validated combination warranting further clinical testing.

## CONFLICT OF INTERESTS

MS is the founder of HKG Epitherapeutics and Montreal EpiTerapia. DC is also engaged by HKG Epitherapeutics and Montreal EpiTerapia. All other authors declare no competing financial interests.

## AUTHOR CONTRIBUTION

**Niaz Mahmood:** Data curation (equal); Formal analysis (equal); Methodology (equal); Validation (lead); Visualization (lead); Writing‐original draft (equal); Writing‐review & editing (equal). **Ani Arakelian:** Data curation (supporting); Formal analysis (supporting); Methodology (supporting); Validation (supporting). **David Cheishvili:** Formal analysis (supporting); Software (supporting); Writing‐review & editing (supporting). **Moshe Szyf:** Conceptualization (equal); Funding acquisition (equal); Resources (supporting); Software (supporting); Supervision (supporting); Writing‐review & editing (equal). **Shafaat A. Rabbani:** Conceptualization (equal); Funding acquisition (equal); Investigation (lead); Project administration (equal); Resources (lead); Supervision (lead); Writing‐original draft (equal); Writing‐review & editing (equal).

## ETHICAL APPROVAL

All in vivo procedures were performed in compliance with the protocol assessed and approved by the McGill University Facility Animal Care Committee (FACC).

## Supporting information

Appendix S1

Appendix S2

Appendix S3

## Data Availability

All data analysed or generated during this study are either available within the main article or attached as Appendices S1‐S3.
