# Supplementary material for: S‐adenosylmethionine in combination with decitabine shows enhanced anti‐cancer effects in repressing breast cancer growth and metastasis
Source: J Cell Mol Med. 2020 Jul 28;24(18):10322–37. doi: 10.1111/jcmm.15642 (PMC7521255; doi:10.1111/jcmm.15642)
Supplement: Supplementary file 1 — Appendix S1 [file JCMM-24-10322-s001.pdf]

Supplementary Table S1: The primers used in this study are listed below[1-16]

Gene Name		Sequences used for qPCR (5'→ 3')
MUC1	For<br>Rev	CTGCTCCTCACAGTGCTTACAGTTG<br>TGAACCGGGGCTGTGGCTGG
MCL1	For<br>Rev	CCAAGAAAGCTGCATCGAACCAT<br>CAGCACATTCCTGATGCCACCT
DUSP1	For<br>Rev	GGCCCCGAGAACAGACAAA<br>GTGCCCACTTCCATGACCAT
KLF4	For<br>Rev	GTGCCCCGAATAACAGCTCA<br>TTCTCACCTGTGTGGGTTCG
SOX4	For<br>Rev	CCAAATCTTTTGGGGACTTTT<br>CTGGCCCCTCAACTCCTC
ITPR3	For<br>Rev	TATGCAGTTTCGGGACCACC<br>TGCCCTTGTACTCGTCACAC
AGRN	For<br>Rev	CCTGACCCTCAGCTGGCCCT<br>AGATACCCAGGCAGGCGGCA
TSPYL2	For<br>Rev	AGGCACTGGAGGATATTCAG<br>GAAGGGTCTTCGCATCTGGAT
TNC	For<br>Rev	AAGTGAACCTGTCTCAGGGTCATT<br>GCTGTCACCAGGCCAGATG
CADM1	For<br>Rev	ATGGCGAGTGTAGTGCTGC<br>GATCACTGTCACGTCTTTCGT
FADS2	For<br>Rev	ACAAGGATCCCGATGTGAAC<br>TTCGTGCTGGTGATTGTAGG
TFPI2	For<br>Rev	GTCGATTCTGCTGCTTTTCC<br>CAGCTCTGCGTGTACCTGTC
HIST1H2AC	For<br>Rev	GACGAGGAGCTCAACAAACTG<br>ACCTGTCAAATCACTTGCCC
HAS2	For<br>Rev	TTATGGGCAGCCAATGTA<br>ACTTGCTCCAACGGGTCT
CST6	For<br>Rev	CAGGGGCGCAGCAGGAGAAG<br>GCCCACGGACCTGAAGTGCC
NEAT1	For<br>Rev	CCAGTTTTCCGAGAACCAAA<br>ATGCTGATCTGCTGCGTATG
VEGF	For<br>Rev	CCTTGCTGCTGCTCTACCTCCAC<br>CCATGAACTTCACCACTTCG
SERPINB2	For<br>Rev	GAAACGCACTTTCGTGGCAG<br>ACAGCTGTGAACTTGGGCAG
GSTP1	For<br>Rev	GAGGACCTCCGCTGCAAATA<br>CAGCAGGGTCTCAAAAGGCT
GAPDH	For<br>Rev	TGCACCACCAACTGCTTA<br>AGAGGCAGGGATGATGTTC

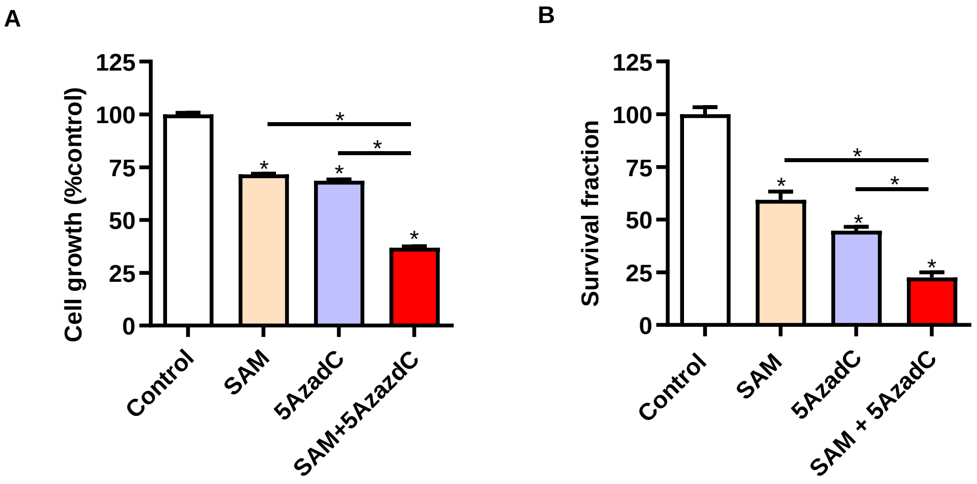

Supplementary Figure S1: Effect of SAM, 5AzadC, and their combination on PyMT-R221A breast cancer cell proliferation and colony formation *in vitro*. **(A)** Murine PyMT-R221A (luminal B subtype) seeded onto 6-well cell-culture grade plates were treated with vehicle only (as control), SAM (200.0 µM), 5AzadC (1.0 µM) and SAM+5AzadC every second day for a period of six days, and the cell proliferation at experimental endpoint was determined through direct cell counting using a Coulter counter. **(B)** At the end of the usual treatment regimen, $5x10^3$ cells from each group were plated in triplicates in each well of six-well plates containing soft agar for a colony assay. The growth medium was replenished every 2-3 days over the next couple of weeks, and the total number of colonies in each well were directly counted under a light microscope and plotted as a bar graph. Results are represented as the mean ± SEM of three independent experiments. Statistical analysis was done using ANOVA followed by *post hoc* Tukey's test, and significant differences are shown by asterisks (*$P < 0.05$).

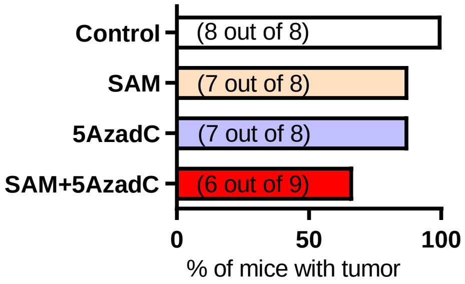

Supplementary Figure S2: Effect of SAM, 5AzadC, and their combination on tumor incidence in NOD-SCID mouse inoculated with MDA-MB-231 cells. While all animals in the control group developed mammary tumors, the percentage of mice with palpable tumors by the experimental endpoint at week 10 markedly decreased in the SAM+5AzadC combination-treated groups. Such an effect on reduced tumor incidence in the combination arm was better than either of the monotherapy treated arms.

Supplementary Table S2: The toxicity profile of the different therapeutic regiment used *in vivo* was measured through biochemical analyses from blood samples. The results are shown as mean ± SEM from three different mice per group. Here, BUN: Blood urea nitrogen; ALT: Alanine aminotransferase; CK: Creatinine kinase

**Parameter**	**Control**	**SAM**	**5AzadC**	**SAM+5AzadC**
Total protein (g/L)	39 ± 0.82	41 ± 0.94	41.67 ± 1.36	45 ± 0.47
Albumin (g/L)	20.67 ± 0.72	21 ± 0.82	22.67 ± 0.27	23 ± 0.47
Glucose (mmol/L)	15.11 ± 0.83	12.93 ± 0.96	15.33 ± 1.4	10.07 ± 0.54
BUN Urea (mmol/L)	6.93 ± 0.25	5.53 ± 0.19	5.7 ± 0.39	7.23 ± 1.08
Creatinine (µmol/L)	10.33 ± 0.27	9.67 ± 0.54	10 ± 0.47	12.33 ± 1.96
Total Bilirubin (µmol/L)	4.67 ± 0.54	3.67 ± 0.54	4.67 ± 0.27	4.67 ± 0.72
ALT (U/L)	35 ± 1.89	29.67 ± 1.19	31 ± 1.25	38.33 ± 3.95
CK (U/L)	306 ± 109.7	349 ± 128.55	450 ± 148.14	435.67 ± 150.18
Sodium (mmol/L)	156.67 ± 3.84	152.67 ± 2.23	152.33 ± 1.91	152.33 ± 0.72
Chloride (mmol/L)	115.33 ± 1.36	118 ± 1.7	116 ± 0.82	115.67 ±1.09

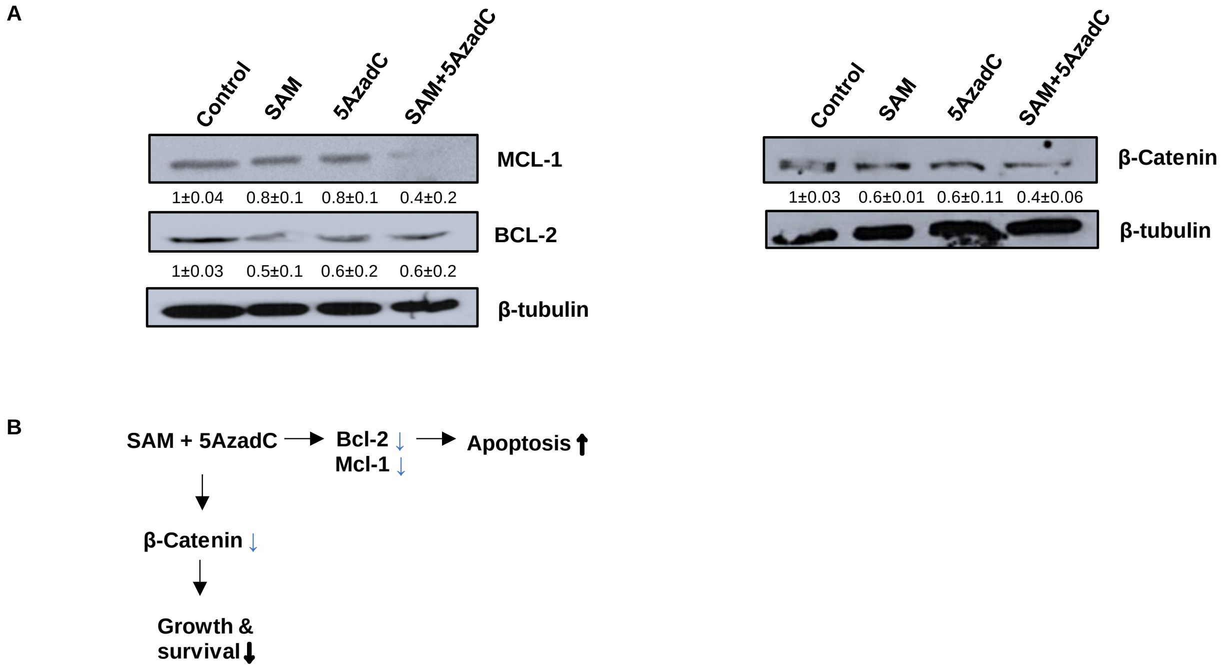

Supplementary Figure S3: Effect of SAM, 5AzadC, and their combination on pro-proliferative and anti-apoptotic factors. (A) Representative western blot of control, SAM, 5AzadC, and combination-treated MDA-MB-231 cells using antibodies against MCl-1, BCl-1, β-Catenin proteins. The β-tubulin expression was used as a loading control and normalization of the samples during band intensity measurement. The normalized densitometric value of the bands were measured by ImageJ (Fiji plugin) and shown as mean ± SEM of two independent experiments. **(B)** Schematic diagram of the functional pathways affected by the SAM+5AzadC combination.

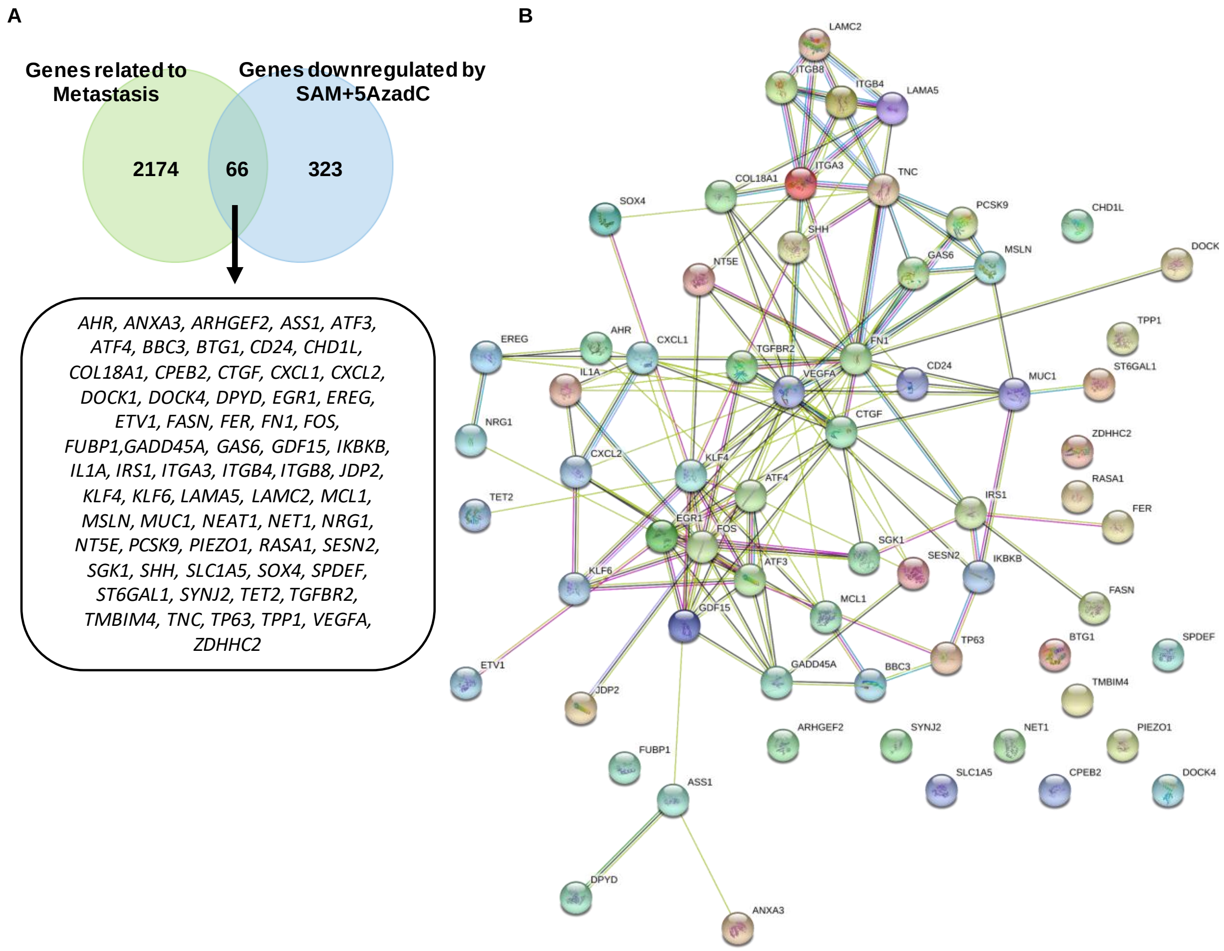

Supplementary Figure S4: (A) Comparison of the significantly downregulated genes upon SAM+5AzadC treatment with the list of metastatic genes obtained from the human cancer metastasis database showed a significant overlap of 66 genes. **(B)** A string network analysis was performed from the genes that showed overlap in **(A)**.

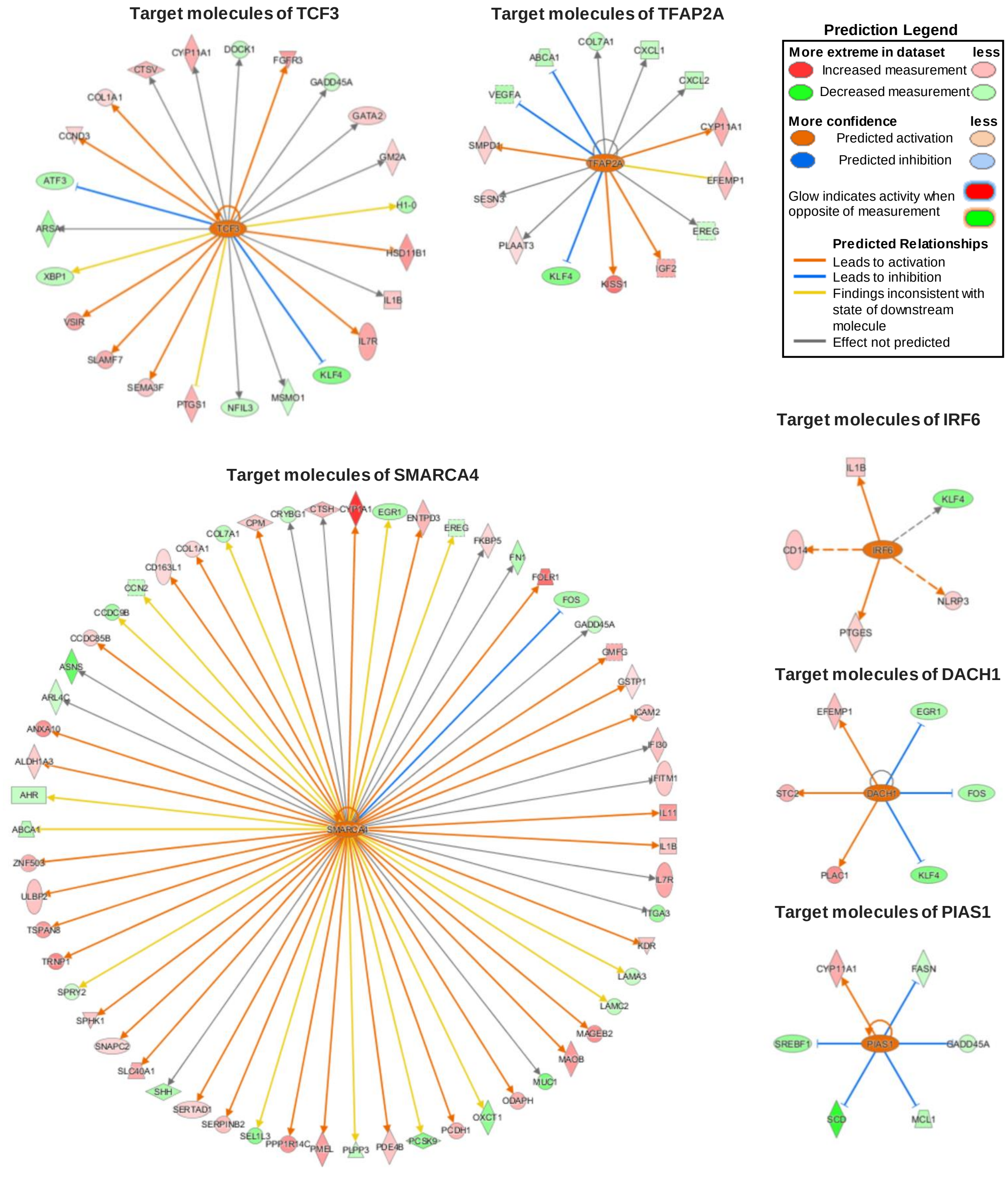

Supplementary Figure S5: Target molecules of the upstream transcription regulators that are significantly activated by SAM+5AzadC treatment according to the IPA tool.

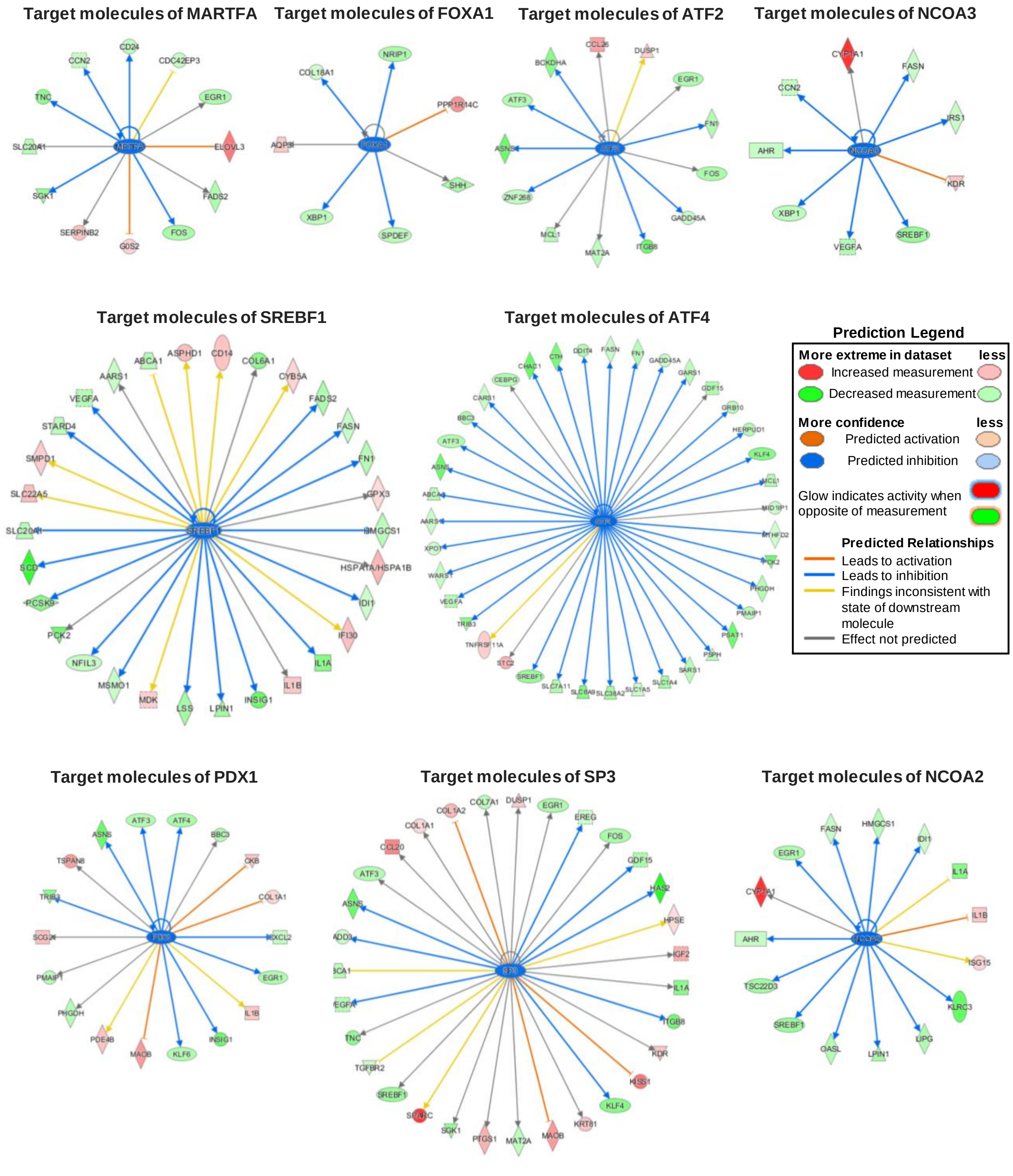

Supplementary Figure S6: Target molecules of several upstream transcription regulators that are significantly inhibited by SAM+5AzadC treatment according to the IPA tool.

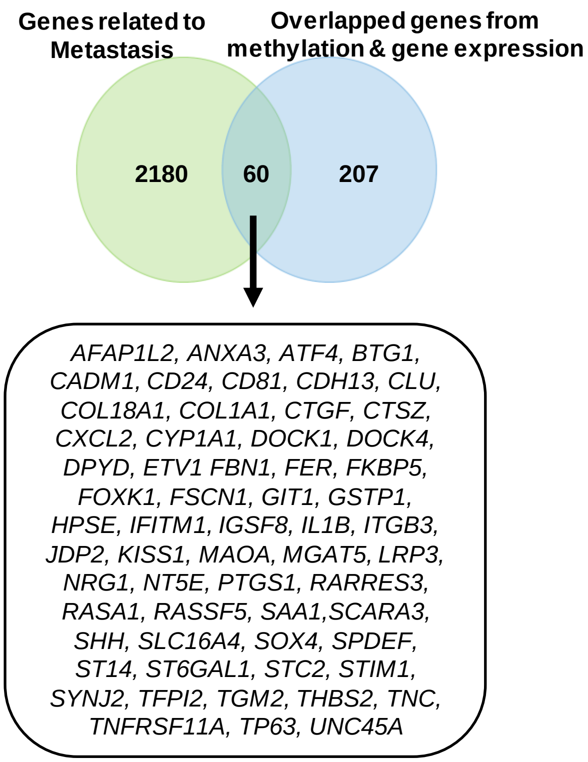

Supplementary Figure S7: Comparison of the 267 genes obtained from the integrative analysis of DNA methylation and RNA expression (in SAM+5AzadC group) with the list of metastatic genes obtained from the human cancer metastasis database showed a significant overlap of 60 genes.

Supplementary Table S3: List of overlapped genes that showed significant changes in the promoter methylation-mediated alteration of gene expression

**Genes with promoter hypomethylation-mediated upregulation**		**Genes with promoter hypermethylation-mediated downregulation**
TNFRSF11A	*LOC101927476*	*PHGDH*
PRSS35	*CYP1A1*	*NRIP1*
NIPAL3	*CTSZ*	*LZTFL1*
NINJ1	*LGMN*	*UFSP2*
AFAP1L2	*SCAND1*	*ACTR1B*
OSGIN1	*MAP1S*	*TRNT1*
TFPI2	*RARRES3*	*SEC31B*
SHROOM3	*KISS1*	*CALHM2*
VTN	*CAPN5*	*NAPRT*
LYPD6B	*FAM8A1*	*ANKRD29*
DDAH1	*MAP6D1*	*TMEM67*
SCG2	*MAP2K3*	*CAB39L*
CD81	*SLAMF7*	*SLC16A7*
CCDC106	*HS6ST1*	*SLC29A2*
GSTP1	*MFAP1*	*FADS2*
SECTM1	*MAGEB2*	
SLC16A4	*SH2D2A*	
SAA1	*LY6E*	
LPPR2	*ITM2C*	
SELO	*CHRNA1*	
SCARF2	*TPST2*	
CCND3	*ICAM2*	
EXTL3		

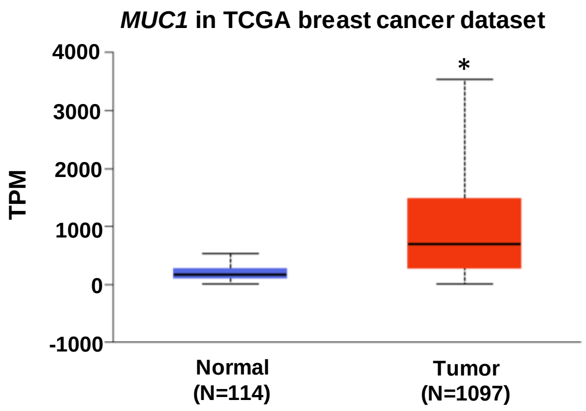

Supplementary Figure S8: The *MUC1* gene expression is significantly elevated in human breast tumor samples relative to the normal samples. Here, TPM: Transcript Per Million.

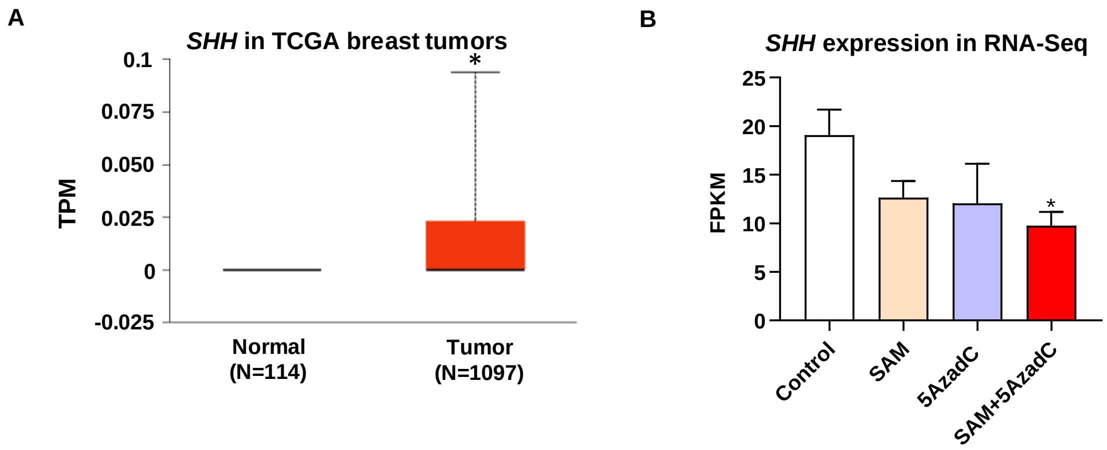

Supplementary Figure S9: The Sonic Hedgehog (*SHH*) gene expression. **(A)** The *SHH* gene expression is significantly elevated in human breast tumor samples relative to the normal samples according to the TCGA database. Here, TPM: transcript per million. **(B)** RNA-Sequencing from the current study revealed that the expression of *SHH* is significantly reduced in the combination setting. Here, FPKM refers to Fragments Per Kilobase of transcript per Million mapped reads.

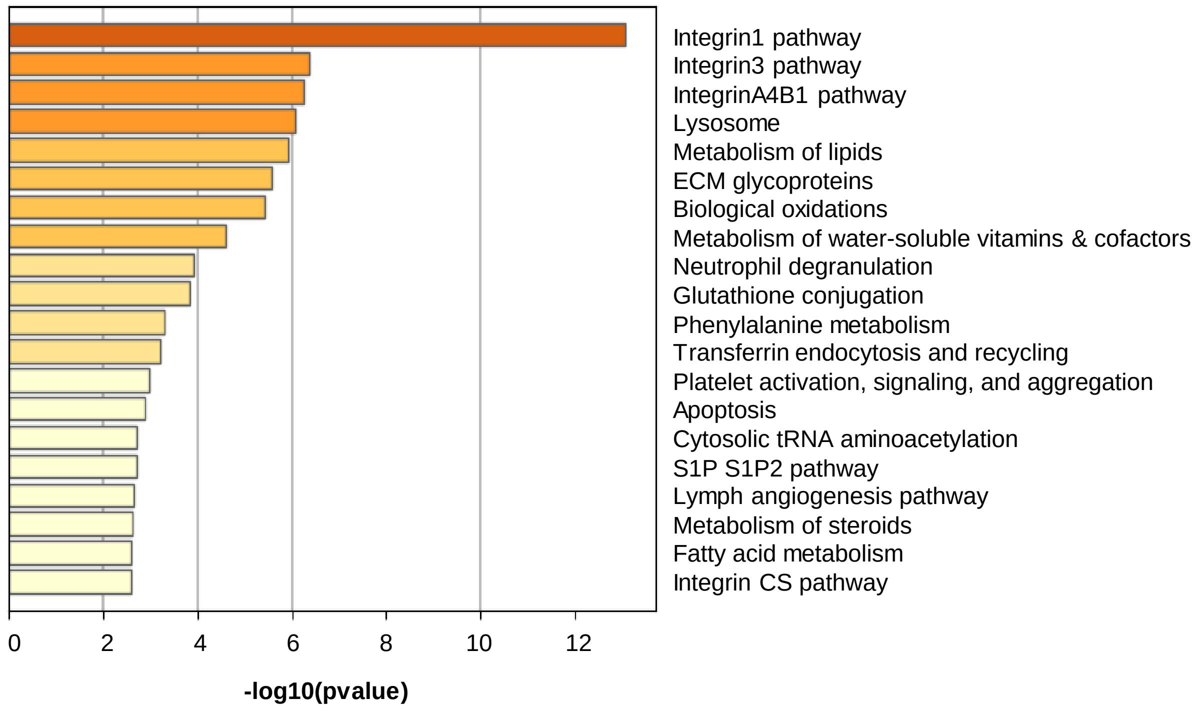

Supplementary Figure S10: Pathways regulated by RNA-Seq obtained unique DEGs in the SAM+5AzadC combination. Several pathways related to invasion and metastasis showed enrichment suggesting their possible regulation by the combination treatment.

References

1. **Gholami O, Jeddi-Tehrani M, Iranshahi M, Zarnani AH, Ziai SA.** Mcl-1 is up regulated by prenylated coumarin, umbelliprenin in jurkat cells. *Iran J Pharm Res*. 2014; 13: 1387-92.
2. **Leyva-Illades D, Cherla RP, Lee MS, Tesh VL.** Regulation of cytokine and chemokine expression by the ribotoxic stress response elicited by Shiga toxin type 1 in human macrophage-like THP-1 cells. *Infect Immun*. 2012; 80: 2109-20.
3. **Riverso M, Montagnani V, Stecca B.** KLF4 is regulated by RAS/RAF/MEK/ERK signaling through E2F1 and promotes melanoma cell growth. *Oncogene*. 2017; 36: 3322-33.
4. **Tiwari N, Tiwari Vijay K, Waldmeier L, Balwierz Piotr J, Arnold P, Pachkov M, Meyer-Schaller N, Schübeler D, van Nimwegen E, Christofori G.** Sox4 Is a Master Regulator of Epithelial-Mesenchymal Transition by Controlling Ezh2 Expression and Epigenetic Reprogramming. *Cancer Cell*. 2013; 23: 768-83.
5. **Sankar N, deTombe PP, Mignery GA.** Calcineurin-NFATc regulates type 2 inositol 1,4,5-trisphosphate receptor (InsP3R2) expression during cardiac remodeling. *J Biol Chem*. 2014; 289: 6188-98.

6. **Eldridge S, Nalesso G, Ismail H, Vicente-Greco K, Kabouridis P, Ramachandran M, Niemeier A, Herz J, Pitzalis C, Perretti M, Dell'Accio F.** Agrin mediates chondrocyte homeostasis and requires both LRP4 and α-dystroglycan to enhance cartilage formation in vitro and in vivo. *Annals of the Rheumatic Diseases*. 2016; 75: 1228-35.
7. **Magni M, Buscemi G, Maita L, Peng L, Chan SY, Montecucco A, Delia D, Zannini L.** TSPYL2 is a novel regulator of SIRT1 and p300 activity in response to DNA damage. *Cell Death & Differentiation*. 2019; 26: 918-31.
8. **Zeeuwen PLJM, de Jongh GJ, Rodijk-Olthuis D, Kamsteeg M, Verhoosel RM, van Rossum MM, Hiemstra PS, Schalkwijk J.** Genetically Programmed Differences in Epidermal Host Defense between Psoriasis and Atopic Dermatitis Patients. *PLOS ONE*. 2008; 3: e2301.
9. **Chen Y, Liu L, Guo Z, Wang Y, Yang Y, Liu X.** Lost expression of cell adhesion molecule 1 is associated with bladder cancer progression and recurrence and its overexpression inhibited tumor cell malignant behaviors. *Oncology letters*. 2019; 17: 2047-56.
10. **Samanta D, Park Y, Andrabi SA, Shelton LM, Gilkes DM, Semenza GL.** PHGDH Expression is Required for Mitochondrial Redox Homeostasis, Breast Cancer Stem Cell Maintenance and Lung Metastasis. *Cancer Research*. 2016: canres.0530.2016.
11. **Vauzour D, Tejera N, O'Neill C, Booz V, Jude B, Wolf IMA, Rigby N, Silvan JM, Curtis PJ, Cassidy A, de Pascual-Teresa S, Rimbach G, Minihane AM.** Anthocyanins do not influence long-chain n-3 fatty acid status: studies in cells, rodents and humans. *The Journal of Nutritional Biochemistry*. 2015; 26: 211-8.
12. **Valdes-Mora F, Song JZ, Statham AL, Strbenac D, Robinson MD, Nair SS, Patterson KI, Tremethick DJ, Stirzaker C, Clark SJ.** Acetylation of H2A.Z is a key epigenetic modification associated with gene deregulation and epigenetic remodeling in cancer. *Genome Res*. 2012; 22: 307-21.
13. **Mino K, Nishimura S, Ninomiya S, Tujii H, Matsumori Y, Tsuchida M, Hosoi M, Koseki K, Wada S, Hasegawa M, Sasaki R, Murakami-Yamaguchi Y, Narita H, Suzuki T, Miyata N, Mizukami T.** Regulation of tissue factor pathway inhibitor-2 (TFPI-2) expression by lysine-specific demethylase 1 and 2 (LSD1 and LSD2). *Bioscience, biotechnology, and biochemistry*. 2014; 78: 1010-7.
14. **Pirngruber J, Shchebet A, Schreiber L, Shema E, Minsky N, Chapman RD, Eick D, Aylon Y, Oren M, Johnsen SA.** CDK9 directs H2B monoubiquitination and controls replication-dependent histone mRNA 3'-end processing. *EMBO reports*. 2009; 10: 894-900.
15. **Chaika NV, Gebregiworgis T, Lewallen ME, Purohit V, Radhakrishnan P, Liu X, Zhang B, Mehla K, Brown RB, Caffrey T, Yu F, Johnson KR, Powers R, Hollingsworth MA, Singh PK.** MUC1 mucin stabilizes and activates hypoxia-inducible factor 1 alpha to regulate metabolism in pancreatic cancer. *Proc Natl Acad Sci U S A*. 2012; 109: 13787-92.
16. **Mahmood N, Cheishvili D, Arakelian A, Tanvir I, Khan HA, Pépin A-S, Szyf M, Rabbani SA.** Methyl donor S-adenosylmethionine (SAM) supplementation attenuates breast cancer growth, invasion, and metastasis in vivo; therapeutic and chemopreventive applications. *Oncotarget*. 2018; 9: 5169.
